# Navigating the Cardiothoracic Publication Landscape: A Primer for Journal Selection

**DOI:** 10.7759/cureus.66970

**Published:** 2024-08-15

**Authors:** Inayat Grewal, Prateek Madaan

**Affiliations:** 1 Radiology, Cleveland Clinic, Cleveland, USA; 2 Internal Medicine, Government Medical College and Hospital, Chandigarh, IND

**Keywords:** article types, publication model, case reports, cardiothoracic, cardiology, impact factor, indexing

## Abstract

Journal selection is very important for any researcher in every field. Publication in a reputable journal not only has a higher reach among a greater number of people but also sets a benchmark for significance and quality. This primer aims to guide researchers in the field of cardiothoracic research, including medicine, surgery, and imaging, to assist in journal selection for their respective articles.

Journal selection depends on a variety of factors, such as impact factors, publication charges, review processes, article types that a journal accepts, and indexing. This primer highlights all these factors in detail that are essential for the selection of a suitable journal. The article emphasizes the importance of these factors in making a reasoned decision about journal selection. This article also focuses on different types of publication models and their implications, including hybrid open access, gold open access, and diamond open access.

In conclusion, this primer aims to provide insights to researchers in the fields of cardiothoracic surgery, medicine, and imaging about the publication landscapes and guide them to strategically plan their submissions.

## Introduction and background

The transition from rigorous research to impactful dissemination of findings is a pivotal step in advancing cardiothoracic surgery and research [1]. While the research process itself demands scientific acumen, the subsequent selection of an appropriate journal for publication presents a unique set of challenges for researchers and clinicians [2]. This primer endeavors to guide researchers through this critical juncture, providing a structured framework for identifying suitable avenues for their work and ensuring that their contributions reach the intended audience [3].

Publication in reputable journals not only amplifies the reach of research but also serves as a benchmark for its quality and significance. This is particularly salient in the field of cardiothoracic surgery, where rigorous, hypothesis-driven research is essential, as emphasized by [4]. It is noted that a significant portion of existing studies suffer from methodological flaws, including a lack of clear, testable hypotheses and issues with generalizability. The process of selecting an appropriate journal for publication is often time-consuming and complex, given the multitude of journals catering to diverse subspecialties within cardiothoracic surgery. This can be especially daunting for early-career researchers [5]. Each journal possesses distinct publication criteria, thematic focuses, and readership demographics, necessitating a thorough evaluation to ensure optimal alignment with a researcher's goals [6].

The strategic selection of a target journal is therefore paramount. It can significantly impact the visibility and overall impact of research findings, underscoring the importance of understanding the nuances of the publication landscape in cardiothoracic surgery. This primer will delve into the multifaceted factors that influence journal selection, encompassing impact factors, indexing, publication model, article types, and review processes. It will also provide a structured approach for assessing these factors, enabling researchers to make informed decisions that maximize the impact and visibility of their work. By addressing the complexities and nuances of journal selection, this primer aims to empower researchers to navigate the publication landscape effectively and contribute meaningfully.

## Review

The role of journal selection in cardiothoracic research dissemination 

The strategic selection of a journal for publishing cardiothoracic research is paramount in ensuring that research findings effectively reach the target audience and achieve maximum impact [7]. The journal chosen not only influences the visibility and dissemination of the research but also significantly impacts the perceived credibility and academic recognition of the authors [8]. A well-aligned journal can serve as a catalyst, amplifying the research's reach, attracting citations, and fostering future collaborations within the scientific community. 

The role of cardiothoracic case reports 

When considering a journal, it is essential to verify if it accepts case reports. Case reports are a vital component of cardiothoracic research by offering detailed accounts of unique clinical scenarios and treatment approaches, thus contributing significantly to medical education and research. They provide valuable learning opportunities for medical students, residents, and seasoned surgeons, shedding light on rare or atypical cases that might not be encountered in routine practice [9]. Unlike other research articles, case reports concentrate on individual patients or small groups, delivering descriptive and observational data about clinical manifestations, diagnostic dilemmas, therapeutic interventions, and patient outcomes [10]. Alsaywid and Abdulhaq provided guidelines for writing case reports, emphasizing their educational value and helping to stay up-to-date as well [11]. Lowenfels et al. highlighted the importance of case reports in graduate medical education, suggesting they contribute to the scientific community despite being considered low in the evidence hierarchy [12]. These reports bridge the gap between theoretical knowledge and practical application, allowing clinicians to glean insights from real-world experiences [13]. While case reports have limitations in terms of generalizability due to their anecdotal nature and potential publication bias, they remain indispensable for knowledge sharing and fostering innovation in the field of cardiothoracic surgery [14].

The selection of the right journal for a cardiothoracic case report is a critical step in ensuring its successful publication and wider dissemination. Authors should prioritize journals that specifically cater to case reports within the cardiothoracic surgery domain, as this ensures that the content aligns with the journal's scope and readership [15]. Additionally, authors should scrutinize the journal's guidelines and publication criteria to ensure that their case report meets all the requirements [7,16,17]. 

Factors like impact factors and indexing status can be considered for gauging the journal's reach and influence within the medical community. Open-access (OA) journals like the Journal of Cardiothoracic Surgery prioritize publishing scientifically sound research and offer wider accessibility to readers compared to subscription-based models. Some journals, like the Journal of Cardiothoracic Surgery and The Cardiothoracic Surgeon, explicitly welcome such submissions, providing a platform for disseminating valuable clinical insights and observations. Authors should also be cognizant of evolving editorial policies and submission guidelines, as these can significantly impact the suitability of a chosen journal [18]. Ultimately, the ideal journal choice will depend on the specific nature and novelty of the article and case reports, making careful evaluation of these factors crucial for successful publication. 

Factors to consider when choosing a journal for cardiothoracic research 

*Journal Indexing and Impact Factor* 

The visibility and impact of your research can be significantly influenced by the journal's indexing status and impact factor. Prioritize journals indexed in reputable databases like PubMed, Scopus, or Web of Science (WoS) for wider dissemination and accessibility. The journal's impact factor, a measure of its average citation rate, serves as a proxy for its influence and reach within the field. Higher impact factor journals often attract a larger audience and may be perceived as more prestigious, potentially leading to increased citations and broader recognition for your work. 

Quartile Ranking 

Within specific subject categories, journals are frequently ranked into quartiles (Q1-Q4), with Q1 denoting the top 25% of journals based on their impact factor. Although not a definitive measure of quality, quartile rankings offer a relative assessment of a journal's standing within its field, aiding researchers in gauging its overall prestige and potential impact. 

WoS and Other Databases 

WoS is a highly regarded comprehensive and authoritative database for research on scientific and scholarly activity that indexes a wide range of scientific journals across various disciplines [19]. Researchers and clinicians can access WoS to find indexed journals, track citation metrics, and assess journal impact factors. WoS offers both free and paid access options, depending on the institution's subscription. Accessing WoS can provide valuable insights into journal metrics and help in making informed decisions about journal selection. 

WoS, a highly regarded citation index, utilizes the International Standard Serial Number (ISSN) to accurately track citations and establish connections between research articles. The ISSN serves as a unique identifier for serial publications, including academic journals. This standardized code is crucial in facilitating the identification, cataloging, and retrieval of publications within various databases. By ensuring accurate and consistent identification, the ISSN not only simplifies the organization of scholarly literature but also aids in assessing the impact and influence of specific publications within the academic community. The ISSN, therefore, plays a pivotal role in upholding the integrity and functionality of citation indexes, contributing to the advancement of research and scholarship across diverse disciplines. 

*Publication Model* 

The landscape of academic publishing is rapidly evolving, with OA gaining prominence. Understanding the publication model is crucial, as it affects the accessibility and cost of publishing. Publication models, including diamond OA, gold OA, subscription, and hybrid OA, determine the accessibility of the published research. Diamond OA journals provide free access to both authors and readers, fostering wider, unrestricted access to research without financial barriers usually funded by institutions or consortia. In contrast, gold OA requires authors to pay an article processing charge, making the research freely available to readers, increasing visibility and accessibility. In the subscription model, access is restricted to subscribers or institutions that purchase subscriptions, which can limit reach, while, in the hybrid OA, authors can choose to pay for OA while other content remains behind a paywall. Learning about different publication models is a must to choose the one that best suits your needs. In the context of cardiothoracic surgery, where rapid dissemination of knowledge can directly influence patient care, OA publishing can be particularly advantageous. However, researchers should be aware of potential drawbacks, such as article processing charges (APCs) associated with gold OA and the limited number of reputable diamond OA journals. Table 1 provides details of various journals and their publication models. 

**Table 1:** Recommended journals for cardiothoracic and related fields WoS: Web of Science; Gold OA: gold open access; Hybrid OA: hybrid open access

Journal name	Indexing original list	Impact factor	Accepts case reports (yes or no)	Publication model	Journal publishing frequency
JACC: Cardiovascular Imaging	Scopus, PubMed/Medline, Abridged Index Medicus, Sci-E, Embase, Elsevier Biobase, Biosis Citation Index	14	No	Hybrid OA (4500 USD)	12
Circulation: Cardiovascular Imaging	PubMed/Medline	7.5	Yes	Hybrid OA (3806 USD)	12
The Annals of Thoracic Surgery	PubMed, WoS	7.2	Yes	Subscription	12
Radiology: Cardiothoracic Imaging	PubMed/Medline, Scopus, WoS	7	Yes	Gold OA 3500	6
Journal of Cardiovascular Magnetic Resonance	PubMed/Medline, Scopus, Sci-E	6.4	No	Gold OA (2990 USD)	1
European Heart Journal: Cardiovascular Imaging	PubMed/Medline	6.2	No	Hybrid OA	12
Journal of Cardiovascular Computed Tomography	PubMed/Medline, Sci-E, Scopus	5.4	Yes	Gold OA (3340 USD)	6
Journal of Cardiothoracic Surgery	PubMed, Scopus	5.2	Yes	Gold OA	12
European Journal of Cardio-Thoracic Surgery	PubMed, Scopus	4.9	Yes	Hybrid OA	12
Journal of Thoracic Imaging	Sci-E	3.3	No	Hybrid OA (2833 USD)	6
CardioVascular and Interventional Radiology	Medline, Scopus, Sci-E	2.9	Yes	Hybrid OA (4190 USD)	12
Journal of Nuclear Cardiology	Medline, Scopus, Sci-E, Embase	2.4	Yes (case series)	Hybrid OA price	6
International Journal of Cardiovascular Imaging	Medline, Sci-E, Scopus	2.1	Yes	Hybrid OA (3590 USD)	12
Current Cardiovascular Imaging Reports	Google Scholar, Embase, Inis Atomindex, Sci-E, Scopus, Scimago	0.9	Yes	Hybrid OA (3890 USD)	12

Publication Frequency 

Understanding the journal's publication frequency helps gauge the timeliness of dissemination. Journals with more frequent publication schedules offer faster dissemination of research findings, which is especially important for researchers looking to quickly share their findings with the community. For example, the Journal of Cardiothoracic Surgery publishes monthly, providing timely updates to the field. Table 1 provides the publication frequency of the most cited cardiothoracic journals. 

Besides the aforementioned factors, other aspects like the journal's target audience, article types, and peer-review process should also be taken into account. Aligning your research with the journal's target audience ensures that your findings reach the most relevant readers and potential collaborators. Evaluating the journal's typical article types helps determine if your work fits within their publication scope. Additionally, understanding the journal's peer-review process can provide insights into the time frame and rigor involved in getting your research published. By meticulously considering these factors, researchers can make informed decisions when selecting a journal, ultimately enhancing the impact and visibility of their cardiothoracic research.

Pre-submission strategies to optimize journal selection for cardiothoracic research manuscripts 

The dissemination of scientific research through publication is a critical step in advancing knowledge and improving clinical practice, especially in cardiothoracic research, encompassing both cardiac and thoracic surgery disciplines. To ensure that research findings reach the intended audience and achieve maximum impact, careful consideration must be given to the selection of an appropriate journal for submission. This process, while often overlooked, can significantly influence the visibility, credibility, and overall success of a research publication. 

Before submitting a manuscript, several key factors warrant meticulous attention. First and foremost, authors should thoroughly review the scope and aims of the target journal to ensure alignment with their research focus. This strategic alignment increases the likelihood of acceptance, as it demonstrates that the research contributes to the journal's specific area of interest. Equally important is adherence to the journal's author guidelines. These guidelines provide detailed instructions regarding manuscript preparation and submission, including formatting, referencing, and ethical considerations. Failure to comply with these guidelines can lead to unnecessary delays or rejection, highlighting the importance of meticulous attention to detail. Assessing the review process and time to publication is also crucial. Different journals have varying timelines for peer review and publication, which may be a deciding factor for authors with specific deadlines or time constraints. Evaluating publication fees is equally important, particularly for OA journals, as these fees can vary significantly and may need to be factored into research budgets. 

Finally, authors are advised to exercise caution when considering predatory journals, which prioritize financial gain over academic rigor and integrity. Verifying a journal's reputation through indexing status, editorial board credentials, and affiliations with reputable organizations like the Directory of Open Access Journals (DOAJ) or the Committee on Publication Ethics (COPE) can help identify and avoid such predatory practices. 

Cardiothoracic surgery journals 

The literature on cardiothoracic surgical education has underscored a pressing need for enhanced research quality to bolster educational practices and outcomes. Cardiothoracic surgery is a complex field with various subspecialties, each requiring dedicated research and knowledge dissemination. Fortunately, numerous journals cater to these distinct areas, providing platforms for researchers and clinicians to share their findings and insights. 

Key journal categories in cardiothoracic surgery include pediatric surgery, adult cardiothoracic surgery, and cardiovascular imaging (Tables 1-3).

**Table 2:** Journals of cardiology

Journal name	Focus	Target audience
International Journal of Cardiology	All aspects of cardiovascular disease, including original clinical studies	Healthcare professionals involved in cardiovascular research and practice
Journal of Cardiology	Research in the field of valvular heart disease	Researchers and practitioners specializing in valvular heart disease
American Journal of Cardiology (AJC)	Practical, clinical approach to the diagnosis and treatment of cardiovascular diseases	Clinicians involved in the diagnosis and treatment of cardiovascular diseases
Indian Journal of Clinical Cardiology	Development of academic, research, and expression among fellows, researchers, institutes, and hospitals in cardiology	Cardiology fellows, researchers, institutes, and hospitals in India
Cardiology Journal	Publishes original papers, review articles, case reports, short communications, and letters to the editor covering various aspects of cardiology	Cardiologists, researchers, and other healthcare professionals interested in cardiology

**Table 3:** Cardiothoracic international journals

Journal name	Focus	Impact factor	Target audience	Accepts case reports	Publication model	Price estimate	Specific to cardiology
European Journal of Cardio-Thoracic Surgery	All aspects of adult and pediatric cardiac and thoracic surgery	4.9	Cardiothoracic surgeons, anesthesiologists, cardiologists, chest physicians, and allied health professionals	Yes	Hybrid open access	APC (3273 EUR)	No (cardiothoracic surgery)
The Journal of Heart and Lung Transplantation	Clinical and research aspects of heart and lung transplantation	6.863	Transplant physicians and surgeons, researchers, and allied health professionals	Yes	Subscription/hybrid open access	Subscription fee or APC	Yes
Asian Cardiovascular and Thoracic Annals	Research relevant to the Asia-Pacific region	0.7	Cardiothoracic surgeons, cardiologists, and researchers in the Asia-Pacific region	Yes	Hybrid open access	APC (3000 USD)	Yes

Pediatric surgery focuses on the unique challenges and advancements in surgical interventions for children with heart diseases. Prominent journals in this area are Recent Advancements in Pediatric Cardiothoracic Surgery, a special issue within the Journal of Clinical Medicine that serves as a platform for discussing cutting-edge treatments, diagnostic techniques, and emerging trends in pediatric cardiothoracic surgery. It emphasizes innovative surgical approaches, minimally invasive procedures, perioperative care, and long-term outcomes, making it an essential resource for researchers and practitioners in this field. The World Journal for Pediatric and Congenital Heart Surgery also delves into the intricacies of pediatric and congenital heart surgery, encompassing a broad spectrum of topics related to both cardiology and thoracic surgery. 

Adult cardiothoracic surgery covers a wide array of procedures like coronary artery bypass grafting, valve replacements, and aortic interventions. This category is well represented by the Journal of Cardiothoracic Surgery which is an OA journal that serves as a comprehensive resource for research in cardiac, vascular, and thoracic surgery, publishing both clinical and experimental studies. Its OA model facilitates broader dissemination of knowledge, making it accessible to a global audience of researchers and clinicians. The Journal of Cardiothoracic Surgery is also an OA, peer-reviewed platform. It welcomes a diverse range of article types, including research articles, reviews, study protocols, case reports, and editorials. It is a hub for cardiothoracic surgeons, anesthesiologists, cardiologists, chest physicians, and allied health professionals, fostering interdisciplinary collaboration and knowledge exchange. 

With the increasing reliance on advanced imaging techniques in cardiothoracic surgery, journals dedicated to cardiovascular imaging are crucial. They cover echocardiography, magnetic resonance imaging (MRI), and computed tomography (CT) scans and bridge the gap between imaging specialists and cardiothoracic surgeons, enabling a deeper understanding of cardiac anatomy, pathology, and surgical planning. This field is also witnessing a surge in research utilizing artificial intelligence (AI) and machine learning (ML) for tasks like risk prediction, image analysis, and surgical planning. Journals that embrace these cutting-edge technologies are likely to attract high-quality submissions and gain prominence in the coming years. Researchers should consider targeting such journals if their work aligns with these emerging trends. Additionally, the increasing emphasis on patient-reported outcomes and personalized medicine is shaping research priorities, and journals that cater to these areas are likely to be influential. 

Cardiothoracic subspecialties

Cardiothoracic surgery is a broad surgical field encompassing the heart, lungs, esophagus, and other organs within the chest. Due to its complexity, it has branched into distinct subspecialties, each focusing on unique patient populations and surgical techniques. Understanding these subspecialties and the journals that publish research in these areas is crucial for both clinicians and researchers. Pediatric cardiac surgery is a subspecialty addressing congenital heart defects and acquired heart conditions in infants and children. These surgeons perform complex procedures to correct structural abnormalities and improve cardiac function in young patients. Key journals in this field include Pediatric Cardiology and Cardiology in the Young, which publish research on diagnosis, treatment, and long-term outcomes in pediatric cardiology and cardiac surgery. Focused on treating heart diseases in adults, adult cardiac surgery is a subspecialty that encompasses a wide range of procedures, such as coronary artery bypass grafting, valve repair or replacement, and heart transplantation. Leading journals in this field include Circulation and The Journal of Thoracic and Cardiovascular Surgery, which feature articles on surgical techniques, clinical trials, and outcomes research relevant to adult cardiac surgery. 

Thoracic surgery specializes in operations on organs within the chest, excluding the heart. They treat conditions like lung cancer, esophageal cancer, and chest trauma. The Annals of Thoracic Surgery and the Journal of Thoracic Oncology are prominent journals in this domain, publishing research on surgical techniques, oncological treatments, and outcomes in thoracic surgery. Vascular surgery deals with diseases of the vascular system, including arteries, veins, and lymphatic vessels. Vascular surgeons perform procedures like aneurysm repair, bypass surgeries, and endovascular interventions. The Journal of Vascular Surgery and Vascular and Endovascular Surgery are key journals in this field, featuring research on vascular diseases, diagnostic techniques, and surgical and endovascular treatments. 

Checklist for journal selection 

**Figure 1:**
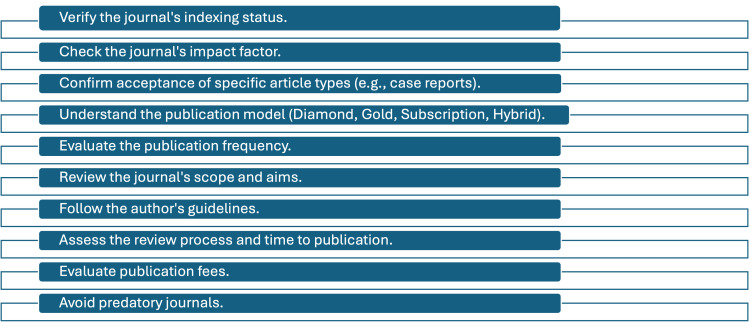
Checklist for journal selection

We have summarized a list of pointers for the researchers that can be used as a checklist to assist in journal selection (Figure 1).

## Conclusions

A comprehensive understanding of the current landscape of cardiothoracic radiology research necessitates familiarity with its key publications. The presented tables in the article serve as a primer, highlighting essential journals for researchers in this domain and offering numerous platforms for researchers and clinicians to publish their work and the ongoing development of cardiothoracic research and medicine. Combining writing strategy training with proper knowledge and study of the journals is crucial for developing high-quality academic texts that meet the coherence and cohesion criteria. However, the informed decision for journal selections saves a lot of hard work and crucial time in the academic tenure.
